# Enhanced XAO: the ontology of *Xenopus* anatomy and development underpins more accurate annotation of gene expression and queries on Xenbase

**DOI:** 10.1186/2041-1480-4-31

**Published:** 2013-10-18

**Authors:** Erik Segerdell, Virgilio G Ponferrada, Christina James-Zorn, Kevin A Burns, Joshua D Fortriede, Wasila M Dahdul, Peter D Vize, Aaron M Zorn

**Affiliations:** 1 Knight Cancer Institute, Oregon Health & Science University, Portland, OR, USA; 2 Division of Developmental Biology, Cincinnati Children’s Research Foundation, Cincinnati, OH, USA; 3 Department of Biology, University of South Dakota, Vermillion, SD, USA; 4 National Evolutionary Synthesis Center, Durham, NC, USA; 5 Department of Biological Science, University of Calgary, Calgary, AB, Canada

**Keywords:** Anatomy, Bioinformatics, Data annotation, Developmental biology, Embryology, Model organism database, Ontology, *Xenopus*

## Abstract

**Background:**

The African clawed frogs *Xenopus laevis* and *Xenopus tropicalis* are prominent animal model organisms. *Xenopus* research contributes to the understanding of genetic, developmental and molecular mechanisms underlying human disease. The *Xenopus* Anatomy Ontology (XAO) reflects the anatomy and embryological development of *Xenopus*. The XAO provides consistent terminology that can be applied to anatomical feature descriptions along with a set of relationships that indicate how each anatomical entity is related to others in the embryo, tadpole, or adult frog. The XAO is integral to the functionality of Xenbase (http://www.xenbase.org), the *Xenopus* model organism database.

**Results:**

We significantly expanded the XAO in the last five years by adding 612 anatomical terms, 2934 relationships between them, 640 synonyms, and 547 ontology cross-references. Each term now has a definition, so database users and curators can be certain they are selecting the correct term when specifying an anatomical entity. With developmental timing information now asserted for every anatomical term, the ontology provides internal checks that ensure high-quality gene expression and phenotype data annotation. The XAO, now with 1313 defined anatomical and developmental stage terms, has been integrated with Xenbase expression and anatomy term searches and it enables links between various data types including images, clones, and publications. Improvements to the XAO structure and anatomical definitions have also enhanced cross-references to anatomy ontologies of other model organisms and humans, providing a bridge between *Xenopus* data and other vertebrates. The ontology is free and open to all users.

**Conclusions:**

The expanded and improved XAO allows enhanced capture of *Xenopus* research data and aids mechanisms for performing complex retrieval and analysis of gene expression, phenotypes, and antibodies through text-matching and manual curation. Its comprehensive references to ontologies across taxa help integrate these data for human disease modeling.

## Background

The embryological literature for *Xenopus*, the African clawed frog, reaches back more than a century [1]. As many forms of human disease are associated with defects in genes involved in the earliest steps of embryonic development, studying the orthologous genes in *Xenopus laevis* and *X. tropicalis* as model systems to elucidate the molecular and cellular pathways through which these genes function has grown in strength in recent decades. Annotation and assembly of the *Xenopus tropicalis* genome demonstrated that it has long regions in which genes exhibit remarkable synteny with the human genome [2], establishing it as an important model for comparative genomics and modeling human gene function. Despite being allotetraploid, *X. laevis* also reflects genetic synteny to humans with full chromosomal duplication of *X. tropicalis* gene sequences. The *X. laevis* genome is currently being assembled and annotated [3]. In addition to being excellent genetic models, both frog species have large externally developing embryos with rapid embryogenesis, allowing easy study of early vertebrate development from fertilization through organogenesis and limb development. Likewise, large experimentally malleable oocytes, particularly from *X. laevis*, are a key tool in studies of ion channel physiology and toxicology and the cell cycle [4]. Oocytes and synchronously developing embryos are easily obtained in large numbers allowing researchers to quickly gather large amounts of data. Together these two *Xenopus* species accelerate our understanding of the mechanisms underlying human health and disease [5], yet a daunting challenge remains: to organize, integrate, and make accessible vast quantities of information as it emerges. Xenbase (http://www.xenbase.org), the *Xenopus* biology and genomics database [6,7], integrates diverse data from high-throughput screens, scientific literature, and other databases (such as NCBI) into a number of database modules, thus allowing researchers to investigate specific genes using well-defined terminologies that bridge different kinds of data. To this end, the *Xenopus* Anatomy Ontology (XAO) was developed as a structured, controlled terminology that 1) unites anatomy and development of the vertebrate embryo with the molecular and cellular research findings, 2) enables powerful data searches, and 3) facilitates accurate annotation of research findings. From its inception, we intended the XAO to be integral to the functionality of Xenbase, and as such the XAO acts as a platform to support automated and manual curation and to power the gene expression search feature.

The XAO provides consistent terms for 1313 anatomical features and developmental stages and draws a detailed conceptual picture of the frog from unfertilized egg to adult. Thousands of relationships between terms describe which tissues are components of other tissues, structures, and anatomical systems, as well as articulating the tissues’ developmental lineage. The timing of each feature’s embryonic development is framed by references to the community-standard Nieuwkoop and Faber (NF) staging series [8]. The XAO is frequently updated and fine-tuned with an emphasis on completeness for each term, and in response to the evolving areas of *Xenopus* research. This is essential to making high-quality annotations and robust database queries of *Xenopus* data with maximal utility to the research community. The implementation of the XAO through Xenbase allows the capture of rich content from the scientific literature and it enables the retrieval and analysis of complex data that have been annotated using the ontology, principally gene expression. Curation of mutant and morphant phenotypes using the XAO is currently in development. The XAO has always been freely available to *Xenopus* researchers and biomedical ontology developers for use in their projects and we continue to encourage them to provide feedback.

Interrelating the XAO with different bio-ontologies has been a key to making *Xenopus* data accessible to the broader scientific and biomedical communities [9], enabling researchers to query across orthogonal biological and human disease databases. We previously reported the initial development of the XAO [10], emphasizing strong representation of embryonic development and interoperability with established species-specific and gross-level anatomy ontologies. In 2009, the XAO was recognized as one of eight exemplar ontologies in the Open Biological and Biomedical Ontologies (OBO) Foundry [11]. We identified at that time several areas of the XAO to be targeted for improvement. It needed expansion to support accurate gene expression curation from the *Xenopus* literature [12] and many existing terms required descriptions and more comprehensive relationships to other terms. Here we report the progress in pursuit of these goals making Xenbase, with its seamless integration of the XAO, a vital and growing biological and genomics database and making the ontology itself a useful resource for *Xenopus* researchers.

## Results and discussion

### Ontology organization and content

Anatomical entities are organized in a single classification (*is_a*) framework in the XAO. Upper-level nodes (e.g., 'compound organ’, 'organism subdivision’) comprise a structural axis of classification cross-referenced to the Common Anatomy Reference Ontology (CARO) [13], providing interoperability with other model organism anatomy ontologies that use CARO as well as a starting point for classifying *Xenopus*-specific features. The XAO reflects various aspects of biological organization with five other logical relationship types.

Cell types, tissues, structures, and sub-systems are described as being *part_of* other tissues, structures, and systems. The lineage of tissues in the course of development is represented by *develops_from* relationships. The timing of their development is indicated by *starts_during* and *ends_during* relationships linking them to specific developmental stages based on the normal table of *Xenopus* development by Nieukoop and Faber [8]. This NF stage series, which has long been the standard in *Xenopus* research, exists as a sub-ontology within the XAO, with *preceded_by* relationships delineating the temporal ordering of the 66 component stages.

The ontology’s developmental tree begins with the specification of the classical vertebrate primary germ layers ('ectoderm’, 'endoderm’, and 'mesoderm’) and branches into the tissues and structures comprising the growing embryo and tadpole. Ultimately, these features are placed within 19 major anatomical systems, from the 'alimentary system’ to the 'urogenital system’. The XAO’s latest release (October 9, 2013) contains 1313 anatomical and developmental stage terms, 5148 relationships, and 695 cross-references to other ontologies (Table 1). Throughout the course of our work we have taken care that the ontology adheres to the community conventions and best practices recommended by the OBO Foundry [14].

**Table 1:** A summary of xenopus anatomy ontology content as of October 9, 2013

	
Total terms	1313
Anatomical entities	1217
Developmental stages	96
Definitions	1313
Synonyms	843
Relationship types	6
Relationships	5148
*is_a*	1328
*part_of*	859
*develops_from*	490
*starts_during*	1197
*ends_during*	1197
*preceded_by*	77
Ontology cross-references	695

The entry for the 'brain’ [XAO:0000010], for example, not only provides a consistent name for this feature and a logical classification as a 'cavitated compound organ’, its relationship to the term 'central nervous system’ indicates where the brain functions while other relationships indicate that the organ first appears at NF stage 22 from its precursor structure, 'anterior neural tube’. Subsequently, the 'forebrain’, 'midbrain’, and 'hindbrain’, are *part_of* 'brain’. Further divisions relate more regions and structures as *part_of* these three main regions of the brain; for example, the 'forebrain’ has 16 distinct terms, 'midbrain’ has 9 terms, and 'hindbrain’ has 13 terms defined by *part_of* relationships. This finer granularity allows more precise gene expression curation that is demanded by modern research.

### Expansion and improvements

As we began to annotate gene expression reported in the literature and to develop a Xenbase expression search interface (released to the public in 2009) it became clear that usability and annotation quality depend on the ontology having comprehensive sets of terms, definitions, and relationships. Expression queries that include anatomical parameters are designed to draw on relationships in the ontology for their functionality. The Xenbase in-house curation interface restricts terms that can be used for annotations at particular stages based on their developmental timing asserted in the ontology.

**Figure 1:**
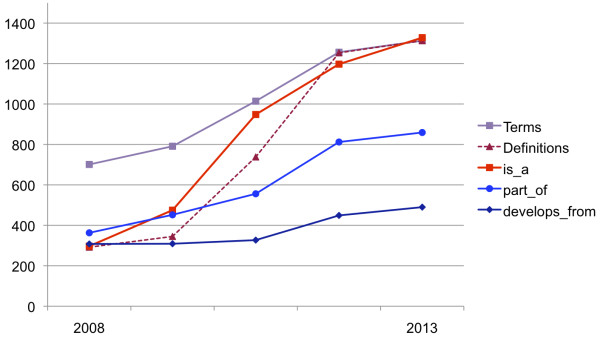
**Growth of the xenopus anatomy ontology.** In the course of its major public releases since 2008, the number of terms in the ontology has grown by 87% and the number of *part_of* and *develops_from* relationships has substantially increased. The majority of terms in the initial release lacked definitions and *is_a* parents, while the latest release (October 9, 2013) is definition- and *is_a*-complete.

**Figure 2:**
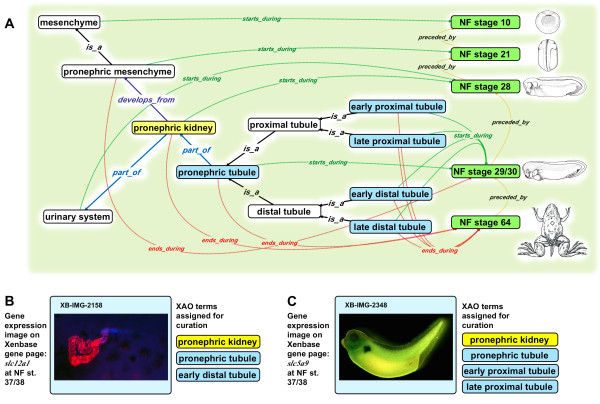
**Inter-relationships of pronephric kidney tissues and structures, their developmental timing, and curated data. A.** The developmental timing of a *Xenopus* anatomical entity (blue fill) is asserted by *starts_during* and *ends_during* relationships to specific embryonic stages (green fill), demonstrated here by the 'pronephric kidney’(yellow fill). **B.** Ontological assertions about developmental timing place constraints on data curation. Expression of the gene *slc12a1* in a NF stage 37/38 embryo (assayed by fluorescent *in situ* hybridization; XB-IMG-2158) is evident in 'early distal tubule’. **C.** Expression of *slc5a1.2* in a NF stage 37/38 embryo (assayed via *in situ* hybridization; XB-IMG-2348) is recorded by curators as taking place in the 'early proximal tubule’ and 'late proximal tubule’. Intermediate NF stages have been omitted for simplicity. Relationship types (colored arrows): *is_a* (black), *part_of* (blue), *develops_from* (purple), *starts_during* (green), *ends_during* (red), *preceded_by* (orange). Images (B) by P. Vize, and (C) by Zhou & Vize [16], used with permission, ©Elsevier (2004).

Since describing the XAO five years ago, we implemented major improvements, expanding it from 701 to 1313 anatomical and developmental stage entities and fleshing out much of its existing content (Figure 1). The ontology, which initially included textual definitions for only 292 terms, now has a definition for every term in the ontology. In its initial release, the ontology comprised to a large extent a “partonomy”, with the principal hierarchical structure depending on which entity each anatomical feature is “a part of” rather than what it is “a type of.” It lacked a single classification framework that fosters the development of good, clear definitions. Concurrent with our effort to be definition-complete, we ensure that every term has an *is_a* parent. Furthermore, the majority of embryonic structures previously lacked specific *starts_during* and *ends_during* stages. All anatomical features now have these relationships following extensive surveys of literature describing various anatomical systems (e.g., the 'skeletal system’ [15]). The ontology has grown to contain 843 synonyms (originally 203), 859 *part_of* relationships (originally 363), and 490 *develops_from* relationships (originally 308). Now, every anatomical term or one of its *is_a* ancestors has at least one *part_of* and at least one *develops_from* relationship to another term. Information that curators glean from the literature has often led to adjustments of start and end stage relationships in the ontology. Ontology-building rules have ensured that the start and end stages associated with related features are consistent and make biological sense. For example, the stage range of 'pronephric mesenchyme’, by rule, must be the same as or fall within that of 'mesenchyme’, its *is_a* parent. Similarly, the validity of *develops_from* and *part_of* relationships is governed by the timing of the related terms; e.g., if 'pronephric mesenchyme’ gives rise to 'pronephric kidney’, the latter must appear sometime within or immediately after the former’s range of NF stage 21–30 (Figure 2).

By using the *starts_during* and *ends_during* developmental stage restrictions we can distinguish transient embryonic structures from tissues that are only present in the adult. Good examples of transient embryonic structures are the 'pronephric kidney’ that *starts_during* NF stage 28 and *ends_during* NF stage 64, and the 'tail region” that *starts_during* NF stage 26 and which is resorbed at metamorphosis and thus *ends_during* 'NF stage 66’. An example of an adult structure is the 'mesonephric kidney’, the kidney in the adult frog, that *starts_during* NF stage 39 and *ends_during* 'death’. Curators validate the temporal constrains of XAO terms as part of their on going annotation of the published literature and adjust start and end stages accordingly. For example during the recent XAO expansion curators found many papers describing the expression of the gene *nkx2.1* as one of the earliest markers of lung fate [17-19]. As a result, the *starts_during* and *ends_during* for the term 'lung primordia’ were revised.

References to other ontologies enable cross-taxon comparisons without the need for complex term translators. The XAO currently contains 695 cross-references; the initial release had only 145. Its close integration with the cross-species Uber Anatomy Ontology (UBERON) [20] provides a bridge to cell types and other vertebrate anatomy ontologies. The XAO’s rich content and referencing has enabled it to be utilized in projects outside of Xenbase, e.g. Bgee [21], which integrates gene expression data from several animal species.

While addressing the XAO’s overall condition, we focused on enhancing several specific aspects of the ontology relevant to *Xenopus* as an animal model:

#### ***Musculoskeletal system***

Integration of amphibian limb phenotypes into the Phenoscape Knowledgebase [22], which links evolutionary phenotypes for vertebrates with data from model organisms [23], prompted improvements in this area. The representation of the 'skeletal system’, which previously comprised only 35 features, grew to include 146 skeletal element and tissue types based on the Vertebrate Skeletal Anatomy Ontology (VSAO) [24]. Of these, 24 are cranial cartilage terms. The ontology now has 45 individual muscle terms, including many cranial muscles. Only three were described in the original XAO release. In addition, we updated the nomenclature and definitions for limb segments ('autopod’, 'stylopod’, and 'zeugopod’) and terms for each digit segment and joint regions in support of phenotype curation.

#### ***Neural crest***

*Xenopus* has proven to be a very important model system for studying neural crest (NC) cell differentiation, stem cell properties, epithelial-mesenchymal transition, and cell migration [25,26]. In order to support the extensive *Xenopus* embryo research in this area we have significantly expanded the XAO NC terms. First, Xenbase curators performed an extensive analysis of the NC literature. After drafting preliminary terms, we consulted domain experts from chick, mouse, fish, and frog communities to improve and coordinate neural crest representation in the XAO. Then, in March of 2012 Xenbase staff participated in a cross-ontology RCN NC workshop [27] where we presented our results and made final term modifications to ensure that the XAO NC definitions were in agreement with other ontologies. This exemplifies our general approach to XAO improvement.

Previously, the main 'neural crest’ functional domains represented in earlier versions of the XAO included only the 'cranial neural crest’, the migrating streams ('mandibular crest’, 'hyoid crest’, and 'branchial crest’) and the 'trunk neural crest’. We expanded this to include 'neural plate border’ (with 'pre-chordal neural plate border’ and 'chordal neural plate border’ domains); 'cardiac neural crest’, 'sacral neural crest’, and 'vagal neural crest’; 'premigratory neural crest cell’, 'migratory neural crest cell’, and 'postmigratory neural crest cell’; and 'anterior branchial crest’ and 'posterior branchial crest’ domains. We built *develops_from* relationships between NC and the tissues and structures to which NC contributes, such as the 'craniofacial skeleton’, 'glial cells’, and 'enteric neurons’ of the 'hindgut’, and the 'outflow tract’ of the 'heart’.

#### ***Neurological structures and stem cells***

While adding 42 more terms for subdivisions and regions of the brain (e.g., 'rhombomere 1’ to 'rhombomere 8’ of 'hindbrain’) and 8 new neuron types ('motor neuron’, 'interneuron’, etc.), the XAO has also doubled the number of neurological placode terms from 10 to 20. These include the 'facial placode’, 'glossopharyngeal placode’, and 'vagal epibranchial placode’ [28]. Stem cell populations of specific regions such as the 'ciliary marginal zone’ of the 'retina’ have also been added.

#### ***Pronephric kidney***

Embryonic kidneys are an important model system for investigating principles regulating multicomponent complex organs [29,30], and *Xenopus* is prized as a model because of its simplicity and experimental accessibility. Xenbase has a rich complement of annotated expression data for *Xenopus* 'pronephric kidney’ development. The XAO now contains significant updates to the definitions, timing, relationships, and synonyms of all pronephric structures.

#### ***Heart and vasculature***

*Xenopus* has been instrumental in studies of vertebrate heart development and new transgenic lines are being used to investigate the molecular mechanisms and complex gene regulatory networks underlying human congenital heart defects and diseases [31]. As the anatomy of the developing heart and vasculature is described and imaged in finer detail [32], the XAO must expand to capture this detail. So far we have added 19 new heart terms such as 'primary heart field’, 'secondary heart field’ and 'epicardial precursor cell’, and this will be a continued focus for ontology improvement.

#### ***Regenerative structures***

*Xenopus* has emerged as a leading model for tissue regeneration research [33]. Recent additions of 'blastema’ terms specific to the fin, limb, tail, and eye allow curators to capture gene expression involved in normal growth and regeneration.

#### ***Oogenesis stages***

With their large size, *Xenopus* oocytes are amenable to studies of ion channels and transporters [34] as well as maternal gene expression. The XAO staging series incorporates seven new unfertilized egg stages, from 'oocyte stage I’ to 'mature egg’ stage, and oocytes have been added as cell types, enabling curation of gene expression during oogenesis.

### Implementation in Xenbase

Integration of the XAO in Xenbase allows the research community to reap practical benefits without needing expert knowledge of ontologies and their design. It also provides important use cases for how the ontology can be used in research applications in general. The gene expression search interface on Xenbase allows researchers to constrain their queries by a range of stages, narrow queries to a single stage, and/or select anatomy search terms from an autocomplete menu, checkbox panel, or expandable tree. It provides an option to include developmental successor or precursor tissues as search parameters. For example, one can perform an expansive search including data for all derivatives of 'neural crest’. Behind the scenes, the database leverages the *is_a*, *part_of*, and *develops_from* assertions in the XAO to retrieve data annotated not only with the exact selected term(s) but with related ones as well. This is a significant improvement over a general text-matching search of Xenbase content, in which a search for 'heart’, for example, would only look for that precise phrase and might fail to retrieve data annotated with a more specific heart component such as 'endocardium’. Searching Xenbase with the term 'pronephros’ provides another use case: of the 203 image records returned by an ontology-based search for 'pronephric kidney’, 25 are not annotated with that exact term and are instead annotated with 'pronephric duct’, 'nephrostome’, etc. Synonyms in the ontology, furthermore, enable users to choose a search term based on their preferred nomenclature and reduce the chances of an empty result.

A complementary gene expression tool called XenMARK is also available on the web [35]. XenMARK uses an ontology-free annotation system that displays expression images as heat map diagrams projected on embryo schematics. Gene expression queries in XenMARK use coarse-grained 19-term anatomy tags (e.g., 'eye’ and 'retina’), thus in XenMARK end users need no detailed knowledge of anatomy terms, but can find genes expressed in the same 'geographical’ area of an embryo. In contrast, the XAO contains over a thousand precise terms (e.g., 42 terms that are *part_of* the eye with a subset of 23 terms that are *part_of* the retina) so Xenbase users can perform advanced fine-grained expression queries. Xenbase and XenMARK have data sharing agreements and reciprocal links to enable users to move between the sites easily.

**Figure 3:**
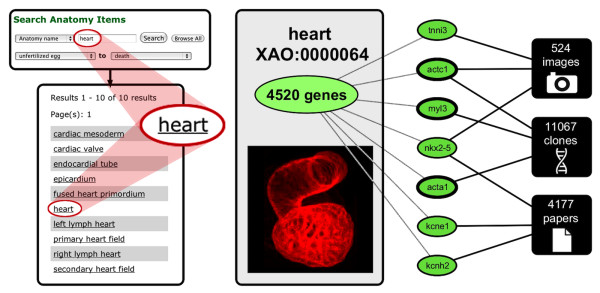
**Links from the Xenopus Anatomy Ontology to *Xenopus *data in Xenbase.** The XAO search anatomy items function returns the exact match on the query term 'heart’. In addition, anatomy features such as 'cardiac mesoderm’ that have relationships to 'heart’ in the ontology are also returned. Clicking on any term takes users to an XAO term summary page. The Expression tab on the 'heart’ term page has links to data related to 4520 expressed genes. Shown schematically is a sample of genes (green fill) that most frequently appear in each of three principal data categories (images, clones, papers: indicated by solid lines). *nkx2-5* expression appears in the greatest number of Xenbase images and is cited in the most publications. The gene *acta1* is associated with the most clones from heart tissue. The genes *actc1*, *myl3* and *acta1* (bold outline) are associated with the greatest combined number of records as of May 20, 2013. The total number of records in each data category for all heart-expressed genes is shown. Heart image by Kolker, Tajchman & Weeks [32], used with permission, ©Elsevier (2000).

To this end, Xenbase provides anatomy and stage term searches [36], taking users to dedicated term summaries, listing term metadata and links to related *Xenopus* information. The Expression tab on every anatomy term page (e.g. 'heart’ [37]) contains links to data related to genes expressed in that tissue or structure, including images with captions, clones, and publication records (Figure 3). Xenbase complements manually curated data with automated and semi-automated annotation processes [6]. The text-mining tool Textpresso [38] processes newly loaded research article metadata from PubMed [39] and captures terms that match anatomy features, thereby providing links from titles, abstracts, and figure captions to XAO terminology.

### Future directions

In the next phase of XAO development we plan to employ the MIREOT (Minimum Information to Reference an External Ontology Term) [40] technique to import upper ontology terms and cell types, replacing our heretofore manual approach, and to make the XAO compliant with the Basic Formal Ontology [41]. We intend to import a “slim” version of the Gene Ontology (GO) [42] cellular component hierarchy, which will allow us to curate gene expression with cellular localization terms. We are currently testing phenotype curation using combined XAO, GO, and Phenotypic Quality Ontology (PATO) [43] terms.

## Conclusions

Thanks to major expansions and improvements, the XAO allows capture of richer content from *Xenopus*-specific scientific literature and research data and provides an essential mechanism for performing complex data retrieval and analysis. We will continue to expand and refine it and to closely interrelate it with other ontologies. The XAO is already essential to gene expression annotation and searches, which will allow researchers to benefit from its further improvement. Xenbase, with an enhanced antibody search function and phenotype database feature currently in development, will continue to drive ontology development. The XAO provides an integral component of entity-quality (EQ) and entity-quality-entity (EQE) annotations made in combination with PATO in order to describe phenotypes. While serving these many functions, the ontology’s comprehensive references to bio-ontologies across taxa will help integrate data that provide insight into human disease, further enhancing the standing of *Xenopus* as an important animal model.

## Methods

The Xenopus Anatomy Ontology is free and open to all users. It may be downloaded from the Xenbase FTP site [44] in file formats that allow it to be opened in two freely available tools, OBO-Edit [45] and Protégé [46]. An OBO-compliant version is available at the OBO Foundry [47] and in Google Projects [48]. Users may also browse the ontology at Xenbase [49] and at a variety of external informatics sites such as Ontobee [50].

Xenbase employs an in-house system of shared documents and spreadsheets where curators request new terms with metadata (definitions, relationships, and stages) and discuss wider structural changes. Public requests and feedback may be submitted at the XAO SourceForge [51] and Google Projects issue trackers.

We regularly seek expertise and input from developers of other vertebrate anatomy ontologies (e.g., the Amphibian Anatomy Ontology [52] and Zebrafish Anatomy Ontology [53]) and the Uber Anatomy Ontology. The XAO adheres to a comprehensive referencing (*xref*) scheme. This consists of CARO *xrefs* for upper-level terms and Cell Type (CL) [54] or UBERON *xrefs* for other applicable terms (the Amphibian Anatomy Ontology, an effort closely related to the *Xenopus* ontology, and the VSAO have recently been absorbed by UBERON, so unlike in previous releases, XAO terms now refer to the relevant UBERON entries and not the original ontologies). We strive to make definitions consistent with UBERON, other anatomy ontologies, and the CL, augmenting them as necessary to reflect their specificity to *Xenopus*.

## Competing interests

The authors declare that they have no competing interests.

## Authors’ contributions

ES, VGP, CJZ drafted the manuscript. ES, VGP, CJZ, KAB, JDF and WMD developed and updated the ontology. PDV and AMZ supervised the project and oversaw XAO implementation. All authors read and approved the final manuscript.
